# Successful Endoscopic Ethanol Injection and Clipping Treatment of Ruptured Duodenal Varices

**DOI:** 10.5152/tjg.2025.25502

**Published:** 2025-11-21

**Authors:** Bahri Abayli, Yeliz Simsek, Begum Seyda Avci, Adnan Kuvvetli, Akkan Avci, Cigdem Erhan

**Affiliations:** 1 Department of Gastroenterology and Hepatology, Seyhan State Hospital, Adana, Türkiye; 2 Department of Emergency Medicine, Adana City Research and Training Hospital, Health Science University, Adana, Türkiye; 3 Department of Internal Medicine, Adana City Research and Training Hospital, Health Science University, Adana, Türkiye; 4 Department of General Surgery, Adana City Research and Training Hospital, Health Science University, Adana, Türkiye; 5 Department of Internal Medicine, Seyhan State Hospital, Adana, Türkiye

To the Editor,

Collaterals developing in parts of the gastrointestinal tract other than the esophagus and stomach are called ectopic varices. The duodenum is the most common site of ectopic varices after the rectum.^1^ Duodenal varices (DVs) consist of collaterals between the portal vein and the systemic circulation, and their primary cause is portal hypertension. Treatment of DV bleeding is challenging, with mortality rates approaching 40%. A standardized treatment for DV rupture has not yet been established. Treatment options include endoscopic varix ligation therapy, sclerotherapy, interventional embolization of the feeding vessels or portosystemic stent shunts, and surgery.^1^ These available treatment modalities have been reported mainly as case series in the literature. There is no randomized controlled study on this subject yet.

In the case presented, duodenal varix rupture was successfully treated with ethanol injection followed by clipping. To our knowledge, this is the first case report in which ethanol injection and clipping were used in the treatment of DV rupture.

Written informed consent was obtained from the patient for the publication of this case report.

## Case Presentation

The patient was a 72-year-old man who presented to the emergency department with complaints of abdominal pain, nausea, and bloody vomiting. He had a history of chronic liver disease (cirrhosis) for 5 years and had experienced hepatic encephalopathy once. He had previously undergone band ligation three times for esophageal varices and once for duodenal varix bleeding. He was smoking and drinking alcohol. The patient was informed about his medical history, current findings, and the disease, and written consent was obtained.

On admission physical examination, Glasgow coma score was 15 and vital signs were stable (body temperature of 36.5°C, blood pressure of 110/60 mmHg, heart rate 60 beats/min and oxygen saturation of 97%). The abdomen was slightly distended and splenomegaly was present. Rectal examination revealed melena.

Initial laboratory evaluation revealed hemoglobin level of 8.7 g/dL (reference range: 13.0-17.5), hematocrit of 27% (40%-50%), leukocyte count of 9 × 10^9^/L (4-11 × 10^9^/L), platelet count of 141 × 10^9^/L (150-400 × 10^9^/L), prothrombin time of 11.8 seconds (9-13 seconds), activated partial thromboplastin time of 27.1 seconds (25-35 seconds), and international normalized ratio of 1.1. Liver, renal, and electrolyte parameters were within normal limits.

Abdominal ultrasonography revealed a cirrhotic liver and splenomegaly.

Treatment of the patient was initiated with fluid resuscitation and blood transfusion through wide bore peripheral venous access. Urgent esophagogastroduodenoscopy was performed. Varicose veins were observed in the esophagus and sclerotherapy was performed. Gastroscopy revealed no fundal varices. Diffuse erythema was observed in the antrum and angulus wall and mucosa. Bulbus mucosa was normal and varices were observed in the second portion of the duodenum, opposite to the bulb. Massive bleeding from a ruptured varicose vein in the horizontal part of the dudenum was observed. Hemostasis was achieved by injecting ethanol into the bleeding site. Afterwards, endoscopic clipping was applied to the varicose vein area. No bleeding was observed after the procedure. All endoscopic treatments were performed by an experienced endoscopist. There was no bleeding and hemodynamic instability during follow-up. No decrease in hemoglobin levels was observed. The patient was discharged after 4 days of hospitalization. No bleeding was reported during 1 year of regular follow-up in the gastroenterology outpatient clinic.

## Discussion

Duodenal varices are rare. Rupture is associated with severe hemodynamic instability and high mortality.^1^ Duodenal varices may cause increased bleeding due to portal blood flow, making treatment more difficult. There is no definitive treatment strategy for DVs. Appropriate treatment is selected according to the hemodynamic status of the patient. The goal is to achieve hemostatic stabilization and radical treatment to prevent recurrence.

In the literature, treatment strategies for DVs are still limited to case series. It remains unclear which treatment strategy is superior for DVs.^1^ There are cases in the literature in which sclerotherapy using N-butyl-2-cyanoacrylate injection was successfully used in the treatment of DV bleeding.^2^ In a case report by Seo et al, sclerotherapy with endoscopic injection of ethanolamine oleate was performed for the treatment of DV rupture.^3^ Chen et al^4^ achieved hemostasis by applying cyanoacrylate injection after a metal clip to the ruptured DV. In our case, sclerotherapy was performed with ethanol injection, followed by clip application, and the treatment was successful. While endoscopic alcohol injection proved effective in controlling duodenal varix bleeding in this case, it is important to acknowledge the potential risks and limitations of alcohol injection, including mucosal necrosis, perforation, ulceration, and local pain. Although this method is effective, it is very important to be careful in patient selection and to follow the patient closely. There are randomized controlled studies on the treatment of esophageal and gastric varices with alcohol injection.^5^ But, there is no such study for DV. Since cyanoacrylate, which is commonly used for DV, was not available in our hospital, ethanol injection was applied as an alternative. Patient follow-up is important because hemodynamic deterioration may be observed after DV treatment. No complications were observed during the monthly follow-up of our patient.

This case demonstrates that endoscopic alcohol injection followed by hemostatic clip application can effectively achieve hemostasis in duodenal variceal bleeding. It represents a practical and viable alternative, particularly in settings where cyanoacrylate therapy is unavailable.

## Data Availability

The data that support the findings of this study are available on request from the corresponding author.
